# Evaluating Lactate and D-Dimer as Mortality Predictors of Paediatric Multiple Organ Dysfunction Syndrome: A Prospective Study in a Low-Middle Income Country

**DOI:** 10.21315/mjms2024.31.4.10

**Published:** 2024-08-27

**Authors:** Phuong Minh Nguyen, Khai Viet Tran, Hung Viet Phan, Khai Quang Tran, Duc Long Tran, Huong Thien Mai, Thu Minh Pham Vo, Tho Kieu Anh Pham, Ly Cong Tran

**Affiliations:** 1 Department of Paediatrics, Faculty of Medicine, Can Tho University of Medicine and Pharmacy, Can Tho City, Vietnam; 2 Department of Internal Medicine, Faculty of Medicine, Can Tho University of Medicine and Pharmacy, Can Tho City, Vietnam; 3 Department of Physiology, Faculty of Medicine, Can Tho University of Medicine and Pharmacy, Can Tho City, Vietnam

**Keywords:** multiple organ dysfunction syndrome, lactates, D-dimer, child mortality, intensive care units

## Abstract

**Background:**

Multiple Organ Dysfunction Syndrome (MODS) is a complex medical condition characterised by dysfunction across multiple organs. With limited information available on mortality prediction in the paediatric population, particularly in low-middle income countries, this study evaluates the mortality predicting capabilities of lactate, D-dimer, and their combination.

**Methods:**

This prospective study involved paediatric patients admitted to the paediatric intensive care unit (PICU) of the largest central children’s hospital in the Mekong Delta region, Vietnam, from 2019 to 2021. The discriminative ability and calibration of both individual and combined tests were assessed using the receiver operating characteristic (ROC) curves and the Hosmer-Lemeshow goodness-of-fit test.

**Results:**

Among the patients studied, 63.1% did not survive. Lactate and D-dimer concentrations were significantly higher in the non-survivor group (*P* < 0.001). The area under the curve (AUC) values for lactate, D-dimer and the combined lactate-D-dimer test were 0.742, 0.775 and 0.804, respectively, with the combination showing the highest AUC value, though without statistical significance. Specific thresholds for lactate, D-dimer and the combination yielded sensitivities of 75.5%, 71.7%, and 66.0%, respectively. All three tests showed no statistically significant differences between observed and predicted mortality in the Hosmer-Lemeshow test (all *P-*values > 0.05).

**Conclusion:**

Lactate and D-dimer levels showed a significant association with mortality, along with good discrimination and calibration abilities. These results highlight the utility of lactate and D-dimer as effective predictors in paediatric MODS, particularly in resource-limited settings, and their role in improving patient outcomes.

## Introduction

Multiple Organ Dysfunction Syndrome (MODS) represents the physiological failure of two or more organ systems, encompassing cardiovascular, respiratory, nephrological, hepatic, gastrointestinal, haematologic and neurological systems (1–3). Research in developed countries has found that nearly 75% of non-survivors in intensive care units have MODS identified as the cause of death (4). The prevalence of MODS among children admitted to paediatric intensive care units (PICUs) ranges from 10% to 50% (5). Globally, MODS is recognised as a critical condition that necessitates extended medical management and consumes substantial healthcare resources. Critically ill patients with advanced MODS typically endure three times longer intensive care unit stays and require more respiratory support than those without MODS (2). In developing countries, inadequate healthcare infrastructure exacerbates societal and economic burdens, as reflected in increased mortality rates.

MODS often arises in the final stage of severe sepsis (6). The World Health Organization’s first global report on sepsis underscores the struggle against the millions of deaths and disabilities caused by sepsis, a challenge further compounded by significant knowledge gaps, particularly in low- and middle-income countries (7). This substantial burden highlights the urgent need for early diagnosis and effective prognostic strategies in managing MODS, which are essential for mitigating its severe outcomes across all countries, regardless of income levels.

In exploring mortality predictors for MODS patients, D-dimer and lactate levels have emerged as potential biomarkers. While the D-dimer’s role is highlighted by some ambiguity in the literature (8), lactate levels have been consistently associated with adverse outcomes, suggesting a correlation with mortality in critical conditions, such as shock and MODS (9–11). Notably, elevated lactate levels beyond 2 mEq/L are linked to increased mortality risks (12, 13), with specific studies pointing to a significant escalation in mortality rates at thresholds exceeding 2.55 mmol/L (14). Moreover, the role of D-dimer as a fibrinolysis marker draws attention due to its prognostic implications, in which deviations from normal levels are associated with altered mortality risks in sepsis, a common precursor to MODS (15–17). These findings emphasise the potential of both lactate and D-dimer levels as valuable biomarkers for predicting mortality in paediatric patients with MODS, signifying their role in guiding clinical decision-making and improving patient outcomes.

Numerous studies have explored various paediatric MODS mortality prediction tools, especially scoring systems (18–20). Although single or dual laboratory tests typically exhibit less prognostic efficacy than multi-variable scoring systems, resource limitations in low- to middle-income settings, such as Vietnam, still pose significant challenges in accessing comprehensive evaluations of these complex tools. Therefore, this study aimed to assess the predictive ability of lactate and D-dimer levels on the first day of admission to the PICU for child MODS mortality in the specific context of Vietnam’s healthcare setting.

## Methods

### Study Design and Participants

This research was a prospective cohort study conducted in the PICU of a principal children’s hospital in the Mekong Delta region of Vietnam, spanning April 2019 to June 2021. Serving as a major referral hub for critically ill paediatric patients, the PICU plays a vital role in the region. The study included paediatric patients aged 1 month old–15 years old who fulfilled the MODS diagnostic criteria developed by Proulx (3) upon their arrival in the PICU. The study excluded any paediatric patient who had a PICU stay shorter than 24 h, who died within the first 24 h of being admitted or whose medical data were incomplete.

All patients admitted to the PICU during the study period who met the inclusion criteria and did not meet the exclusion criteria were included in the study. They were followed up until in-hospital death or 24 h post-discharge, whichever occurred first, to document the mortality outcome.

To accurately calculate the required sample size for estimating the mortality rate among children with MODS, we utilised the single proportion estimation formula (21). This calculation was based on a previously reported mortality rate of 70% for children with MODS (22), incorporating a margin of error of 10% and a confidence level of 95% (α = 5%). As a result, a sample size of 81 participants was determined to be sufficient for estimating the mortality prevalence in children with MODS under these conditions.

## Data Collection

Demographic, clinical and paraclinical data were collected at the time of the participants’ admission to the PICU. The collected data, including the independent variables for assessing cardiovascular, respiratory, neurologic, haematologic, gastrointestinal, renal and hepatic systems, were ensured to fully comply with the MODS criteria defined by Proulx (3). The venous plasma lactate and D-dimer levels, used as predictive variables for the mortality outcomes of children with MODS, were also recorded. If the test was not conducted at the time of PICU admission due to objective reasons, such as limited resources at that time, the test results obtained within the first day of admission were acceptably used for further analysis. The patients were followed up until 24 h post-discharge from the hospital to record their mortality outcomes. The primary outcome variable was the mortality (survivors versus non-survivors) of children with MODS, determined by the occurrence of mortality either during the hospital stay or within 24 h after discharge.

## Statistical Analysis

The categorical variables were presented as frequencies and percentages. The quantitative variables with normal distribution were presented as means and standard deviations, while those with non-normal distribution were presented as medians and interquartile ranges. The chi-squared test was used to compare two proportions, and Fisher’s exact test was used where applicable. The *t*-test and the Mann-Whitney U test were employed to compare two quantitative variables with normal and non-normal distributions, respectively.

The combination of lactate and D-dimer variables was assessed using logistic regression analysis for mortality outcomes to determine the coefficient values. The probability model derived from this combination was used to conduct receiver operating characteristic (ROC) curve analysis and to determine the optimal cut-off value and its prognostic performance parameters. The model for the lactate and D-dimer combination variable is as follows: y′ = (−1.252) + 0.192 × lactate + 0.111 × D-dimer, with the probability calculated as 1/(1+e^-y′^).

To assess the validity of lactate and D-dimer, both individually and in combination, ROC curve analysis was utilised for discrimination alongside the Hosmer-Lemeshow goodness-of-fit test for calibration. Discrimination was evaluated by calculating the area under the curve (AUC) and its 95% confidence interval (CI), assessing the ability to differentiate between survivor and non-survivor groups. An AUC of 0.5 suggests no discrimination, 0.7–0.8 is considered good, 0.8–0.9 is considered very good, and greater than 0.9 is considered excellent (23, 24). The DeLong test was used to compare two AUCs. For prognostic performance, the optimal cut-off value was determined using the Youden index and the performance parameters, such as sensitivity, specificity, positive predictive value and negative predictive value, were calculated.

Calibration was assessed using the Hosmer-Lemeshow goodness-of-fit test, which verified the degree of agreement between observed and predicted mortalities, as determined by lactate, D-dimer and their combination. A *P*-value ≥ 0.05 indicated consistency between the predicted and observed values, suggesting good calibration. All analyses were performed using the R 4.1.3 programme.

## Results

### Patient Characteristics

Among the 306 patients admitted to the PICU during the study period, 84 met the inclusion criteria. Among these patients, non-survivors constituted more than 60% (Table 1).

Sepsis was the predominant diagnosis in both the survivor and non-survivor groups, but there was no significant difference in its prevalence between these two groups haematologic (*P* = 0.143). In terms of specific organ systems, the respiratory system was the most common type of organ failure, observed in 98.1% of the non-survivor group and 80.6% of the survivor group. The prevalences of neurological and respiratory system failures were remarkably higher in the non-survivor group than in the survivor group (*P* < 0.05).

In the non-survivor group, the median platelet count was half that of the survivor group, although this difference was not significant. Furthermore, the D-dimer and lactate levels were significantly higher in the non-survivor group than in the survivor group (*P* < 0.001).

### Discriminative Ability for Mortality Prediction

As shown in Table 2, the overall AUC for lactate was 0.742 (95% CI: 0.636, 0.848), indicating good discrimination. Specifically, in individuals over 5 years old, the AUC reached 0.831 (95% CI: 0.662, 1.000; *P* = 0.004). When analysed by gender, the AUC values were 0.769 for males (95% CI: 0.647, 0.892) and 0.692 for females (95% CI: 0.420, 0.963).

For the D-dimer test, the AUC was 0.775 (95% CI: 0.672, 0.878). Notably, a similar trend with approximately the same discrimination AUC was observed in the subgroup analyses, regardless of whether the main diagnosis was with or without sepsis. This trend was also found in both the lactate and D-dimer tests.

In the combined lactate-D-dimer test, the overall AUC of 0.804 (95% CI: 0.710, 0.898) was higher than in the other two tests, suggesting better discrimination in predicting mortality. In terms of gender, the AUC for females was exceptionally high at 0.908 (95% CI: 0.794, 1.000), denoting excellent discrimination.

**Figure 1:**
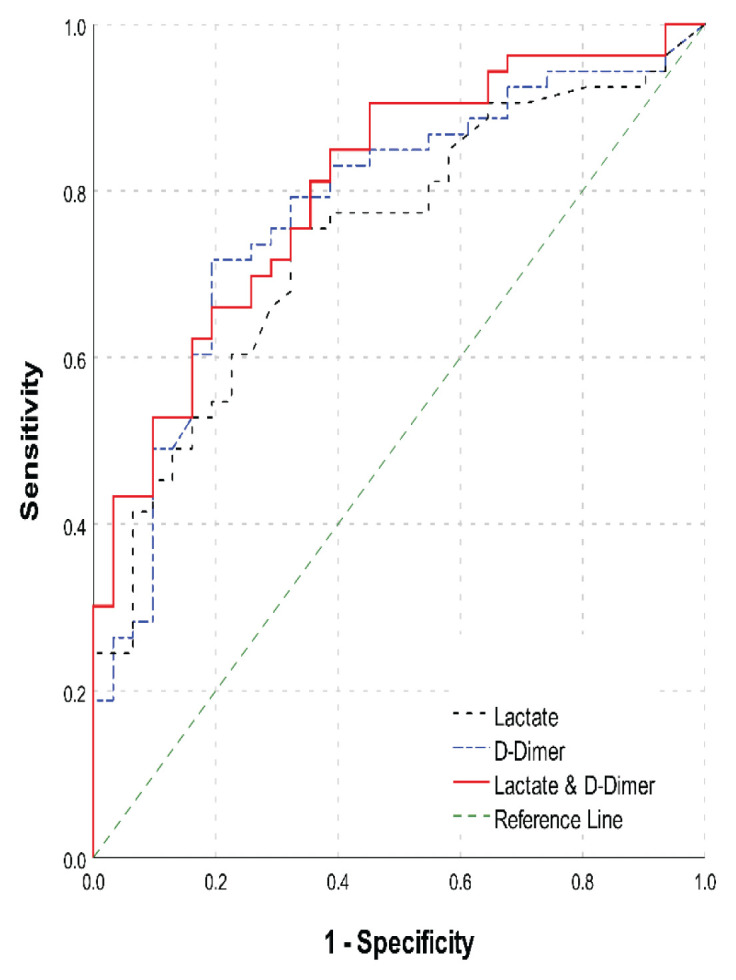
AUC ROC analysis of lactate, D-dimer and the combination variables

In the DeLong test, no significant differences were found when comparing the three tests (*P* > 0.05). This suggests similar efficacy among the tests in predicting mortality. Notably, the difference in AUC between lactate alone and the combined lactate-D-dimer test (*Z* = −1.836, *P* = 0.066) was not statistically significant, as illustrated in Table 2 and Figure 1.

### Prognostic Performance of the Three Tests

Using the Youden index, the optimal cut-off values of lactate, D-dimer and the combined lactate-D-dimer test to predict fatalities were 4.8, 2.4 and 0.592, respectively (Table 3). The lactate test demonstrated the highest sensitivity among the three tests, with a sensitivity (Se) of 75.5% (95% CI: 63.9, 87.1). Conversely, for specificity (Sp), both the D-dimer test and the combined lactate-D-dimer test achieved the highest rate of 80.6% (95% CI: 66.7, 94.6). The positive likelihood ratio (PLR) values for lactate, D-dimer and the combined lactate and D-dimer were 2.34 (95% CI: 1.37, 3.99), 3.7 (95% CI: 1.77, 7.75) and 3.4 (95% CI: 1.62, 7.18), respectively, with D-dimer and the combination demonstrating higher values, suggesting a greater likelihood of mortality.

### Calibration Performance of the Three Tests

As shown in Table 4, there were no statistically significant differences between the observed and predicted mortality rates for any of the tests (lactate: *P* = 0.133; D-dimer: *P* = 0.328; lactate and D-dimer: *P* = 0.941), regardless of whether overall or in specific subgroups, such as age group, gender or main diagnosis.

## Discussion

### General Characteristics

Children diagnosed with MODS are at an elevated risk of mortality, particularly those requiring intensive care (25). In our study, the mortality rate was 63%, consistent with the ranges observed in similar studies in Vietnam (26, 27), but it was higher than that in research conducted globally (14, 28). Notably, the fatality rate among sepsis patients in our study was 70.5% (31 out of 44 patients), surpassing the rates reported in recent studies in Vietnam and internationally (14, 29). This difference may be ascribed to variations in the conditions of the patients. A critical aspect of our study was its focus on patients diagnosed with MODS, in contrast to other studies that included all patients admitted to the PICU.

Our analysis identified respiratory and cardiovascular failures as the most frequent organ failures in the non-survivor group (98.1% and 90.6%, respectively) and the survivor group (80.6% for both). These proportions exceeded those reported by Tran et al. (30) and Tantaleán et al. (31). However, their findings regarding the prevalence of these organ failures concurred with our results.

In our study, sepsis was the predominant diagnosis, accounting for 52.4% (44 of 84 cases) of all cases. This proportion rate is consistent with the findings from the studies of Tantaleán et al. (31) and Wilkinson et al. (32), in which sepsis rates among MODS patients were reported as 47.5% and 55.8%, respectively.

### Discrimination

In our study, we observed that the AUC for lactate was 0.742 (95% CI: 0.636, 0.848), which indicates good discrimination. Specifically, for individuals over 5 years old, the AUC increased to 0.831 (95% CI: 0.662, 1.000; *P* = 0.004), denoting very good discrimination ability. When examining gender differences, the AUC values for males and females were 0.769 (95% CI: 0.647, 0.892) and 0.692 (95% CI: 0.420, 0.963), respectively. The comparatively lower discrimination ability in females, as reflected by a broader 95% CI, could be due to the smaller size of the female sample, given that the male-to-female ratio in the study was approximately 2:1. Lactate concentration as a predictor of mortality has been the subject of extensive research. Gi̇Rgi̇N et al. (14) reported in their ROC analysis that lactate levels are an effective indicator of mortality risk, as evidenced by their AUC of 0.861 (95% CI: 0.79, 0.93; *P* < 0.001). Similarly, another study reported an AUC value of 0.818 for mortality forecasting in critically ill PICU patients (33). These findings affirm the use of lactate concentration in predicting mortality, particularly in children with MODS.

In the D-dimer test, the AUC was 0.775 (95% CI: 0.672, 0.878). This good discrimination ability was consistent across subgroups, irrespective of whether the main diagnosis was sepsis or non-sepsis. Consistent with the findings of Wang et al., who recorded an AUC of 0.77 (95% CI: 0.74, 0.79) in their analysis (8), our study also found that the D-dimer is a reliable predictor of mortality. Further supporting this, additional studies have underscored the very good to excellent discriminatory capability of blood D-dimer in predicting in-hospital mortality in children (34, 35). These investigations collectively suggest that despite variations in participants and methodologies across studies, measuring the D-dimer concentration remains a valuable tool for predicting mortality risk in children, especially those with MODS.

For the combined lactate-D-dimer test, we found an AUC of 0.804 (95% CI: 0.710, 0.898), suggesting very good discrimination in mortality prediction. However, the DeLong test revealed no significant differences among the three tests (lactate, D-dimer and the combined tests), indicating their potential comparability in clinical use.

### Diagnostic Performance

In the present study, we established that a lactate concentration of 4.8 mmol/L could be used as a threshold to assess the likelihood of mortality. This cut-off point is similar to that found by Bai et al. (37) but higher than the 3.2 mmol/L cut-off observed in Kawase’s study (36). At a lactate concentration of 4.8 mmol/L, we observed an accuracy (ACC) of 72.6%, Se of 75.5%, Sp of 67.7%, positive predictive value (PPV) of 80% and negative predictive value (NPV) of 61.8%. These results are in accordance with those of Bai et al. (37), who reported Se, Sp, PPV and NPV of 70% each, using a 4 mmol/L cut-off for lactate concentration at PICU admission to predict in-hospital mortality. Choudhary et al.’s (38) study also found that the optimal cut-off for lactate levels at PICU admission for predicting mortality was ≥ 4 mmol/L, with Se of 57%, Sp of 82%, PPV of 84% and NPV of 51%.

For the D-dimer, we identified a cut-off value of 2.4 μg/mL. At this threshold, the D-dimer concentration demonstrated Se of 71.1%, Sp of 80.6%, PPV of 86.4% and NPV of 62.5%. Cheng et al. (34) found an optimal cut-off of 2.56 μg/mL for in-hospital mortality prediction, with Se of 68% and Sp of 93%. Furthermore, Wang et al. (8) revealed that an optimal threshold of 1.53 μg/mL yields Se of 65% and Sp of 77%. A D-dimer level exceeding 10 times the normal upper limit was indicative of an increased risk of mortality (39).

The combined lactate-D-dimer test presented an optimal cut-off value of 0.592, with an ACC of 71.4%, Se of 66.0%, Sp of 80.6%, PPV of 85.4% and NPV of 58.1%. As shown in Table 3, when comparing the performance parameters of the combined lactate-D-dimer test with those of lactate or D-dimer alone, we found that the combination did not significantly enhance prognostic performance. Some parameters of the combination even showed worse ability. Therefore, these findings suggest that in the current study, the combined lactate-D-dimer value is not a better predictor of mortality compared with D-dimer or lactate levels individually in terms of discrimination ability with prognostic performance.

### Calibration

In assessing the calibration of mortality predictions in children with multiple organ dysfunctions, all three tests (lactate, D-dimer and the combined lactate-D-dimer) demonstrated positive outcomes in calibration tests. Subgroup analysis also revealed good calibration, indicating consistent internal validity across the different groups. These results suggest that these tests are effective in precisely predicting mortality within this specific group. The reliability of these tests in forecasting mortality is further shown by their *P*-values: 0.113 for lactate, 0.328 for D-dimer and 0.941 for the combined lactate-D-dimer test. In their 2020 study, Hayashi et al. (40) also observed good calibration using the Hosmer–Lemeshow test for lactate concentration in predicting in-hospital mortality, as evidenced by a *P*-value of 0.713. Therefore, these three tests are considered suitable for predicting mortality in children with MODS due to the close alignment between the predicted and observed mortalities.

### Limitations

This study has some limitations. First, although this study was conducted at a large central hospital over a period of more than 2 years, it was a single-centre study with a limited patient cohort, all of whom were included in a PICU. As a result, the findings’ applicability to the broader paediatric population and other contexts remains a matter of concern. This highlights the imperative for future research across a variety of multi-centre settings to ensure more robust and generalisable results. Second, our assessment of predictors was focused solely on a single time point—upon admission to the PICU for MODS mortality in children. Future research should validate the specific cut-off points and incorporate repeated measurements to allow for a more dynamic and accurate evaluation.

### Implications for Clinical Practice and Future Research

In low-income countries, where medical facilities are often limited, a convenient and straightforward diagnostic tool can play an important role in predicting mortality. To the best of our knowledge, this study represents an initial effort to evaluate the effectiveness of three specific tests in assessing the likelihood of survival in children with MODS. The findings indicate that all three biomarkers have potential clinical value. However, it is suggested that using one of the two individual tests may be preferable to the combined test, which does not significantly enhance mortality prediction capabilities and can be more resource intensive in settings with limited resources. Nonetheless, further research is essential to comprehensively assess the effectiveness of these biomarkers in predicting mortality outcomes in children with MODS.

## Conclusion

This study established that all three tests are suitable for predicting mortality in children with MODS. Although both the individual tests and their combination show good prognostic value, using one of the individual tests is recommended over the combination, which does not significantly enhance the predictive accuracy for mortality. Moreover, in Vietnam, a low-middle income country with limited resources, the lactate or D-dimer test could be more beneficial due to its time efficiency and straightforward methodology. These tests have demonstrated notable discrimination and calibration, making them economically viable and practical options for predicting mortality in paediatric settings. Further research is essential to enhance the generalisability of lactate and D-dimer use in diverse clinical contexts and to integrate these biomarkers into existing treatment protocols for the improved management of MODS in children.

## Figures and Tables

**Table 1:** Demographic and biologic characteristics of studied patients

Characteristics	Non-survivors (*n* = 53)	Survivors (*n* = 31)	Statistic value	*P*-value
Age group, *n* (%)				
< 5 years old	40 (75.5)	18 (58.1)	2.01 (1)	0.155^a^
≥ 5 years old	13 (24.5)	13 (41.9)		
Gender, *n* (%)				
Male	33 (62.3)	25 (80.6)	2.92 (1)	0.13^a^
Female	20 (37.7)	6 (19.4)		
Main diagnosis, *n* (%)				0.011^b^
Sepsis	31 (58.5)	13 (41.9)	2.14 (1)	0.143^a^
Cardiovascular diseases	8 (15.1)	13 (41.9)	7.51 (1)	0.006^a^
Respiratory diseases	2 (3.8)	3 (9.7)	–	0.353^b^
Other	12 (22.6)	2 (6.5)	3.69 (1)	0.055^a^
Required mechanical ventilation, *n* (%)				
Yes	50 (94.3)	20 (64.5)	10.47 (1)	0.001^a^
No	3 (5.7)	11 (35.5)		
Required vasoactive agents, *n* (%)				
Yes	48 (90.6)	20 (64.5)	7.00 (1)	0.008^a^
No	5 (9.4)	11 (35.5)		
Organ dysfunction, *n* (%)				
Neurology	30 (56.6)	0 (0.0)	24.88 (1)	< 0.001^a^
Respiratory	52 (98.1)	25 (80.6)	–	0.009^b^
Cardiovascular	48 (90.6)	25 (80.6)	–	0.314^b^
Haematology	33 (62.3)	17 (54.8)	0.192 (1)	0.661^a^
Gastroenterology	9 (17)	2 (6.5)	–	0.201^b^
Hepatology	4 (7.6)	1 (3.2)	–	0.647^b^
Nephrology	3 (5.7)	0 (0.0)	–	0.293^b^
GCS, mean (SD)	6.2 (2.8)	11.7 (2.5)	−9.16 (70.31)	< 0.001^c^
Platelet count, × 10^9^/L Median (IQR)	113.0 (22.5–299.0)	257.0 (24.0–403.0)	−1.24	0.214^d^
Leucocyte count, × 10^9^/L Median (IQR)	13.0 (5.5–19.2)	7.1 (4.8–18.0)	−1.09	0.272^d^
Lactate, mmol/L Median (IQR)	7.1 (4.6–13.3)	4.0 (2.6–5.5)	−3.68	< 0.001^d^
D-dimer, μg/mL Median (IQR)	4.7 (1.9–12.2)	1.1 (0.8–2.2)	−4.19	< 0.001^d^

Notes: GCS = Glasgow Coma Scale; SD = standard deviation; IQR = interquartile range.

a Chi-square test for independence, with χ^2^-statistic (df) value;

b Fisher’s exact test;

c independent *t*-test, with *t*-statistic (df) value;

d Mann-Whitney U test, with *Z*-statistic value.

All comparisons were made between the non-survivors and survivors groups

**Table 2:** Discrimination for prediction of mortality with lactate, D-dimer and the combination variables

	AUC	SE	95% CI	*P*-value
**Lactate**				
Overall	0.742^a^	0.054	0.636, 0.848	< 0.001
Age group				
< 5 years old	0.692	0.075	0.546, 0.839	0.020
≥ 5 years old	0.831	0.086	0.662, 1.000	0.004
Gender				
Male	0.769	0.062	0.647, 0.892	< 0.001
Female	0.692	0.138	0.420, 0.963	0.162
Main diagnosis				
Sepsis	0.749	0.083	0.587, 0.912	0.010
Non-sepsis	0.742	0.079	0.588, 0.897	0.009
**D-dimer**				
Overall	0.775^b^	0.053	0.672, 0.878	< 0.001
Age group				
< 5 years old	0.771	0.066	0.642, 0.900	0.001
≥ 5 years old	0.796	0.093	0.614, 0.978	0.010
Gender				
Male	0.762	0.063	0.638, 0.886	0.001
Female	0.833	0.080	0.677, 0.990	0.015
Main diagnosis				
Sepsis	0.777	0.077	0.625, 0.928	0.004
Non-sepsis	0.769	0.079	0.617, 0.920	0.004
**Lactate and D-dimer**				
Overall	0.804^c^	0.048	0.710, 0.898	< 0.001
Age group				
< 5 years old	0.760	0.068	0.627, 0.893	0.002
≥ 5 years old	0.905	0.061	0.785, 1.000	< 0.001
Gender				
Male	0.790	0.059	0.674, 0.906	< 0.001
Female	0.908	0.058	0.794, 1.000	0.003
Main diagnosis				
Sepsis	0.764	0.080	0.608, 0.920	0.006
Non-sepsis	0.843	0.063	0.720, 0.967	< 0.001

**DeLong test comparison:** ^a^ versus ^b^ : *Z* = −0.462, *P*-value = 0.644 ^a^ versus ^c^ : *Z* = −1.836, *P*-value = 0.066 ^b^ versus ^c^ : *Z* = −0.572, *P*-value = 0.568				

Note: AUC = area under the curve; SE = standard error; CI = confidence interval

**Table 3:** Prognostic performance of lactate, D-dimer and the combination for mortality prognosis

Parameters	Lactate	D-dimer	Lactate and D-dimer
Cut-off	4.8	2.4	0.592
ACC%, (95% CI)	72.6 (72.2, 73.1)	75.0 (74.6, 75.4)	71.4 (71.0, 71.9)
Se%, (95% CI)	75.5 (63.9, 87.1)	71.7 (59.6, 83.8)	66.0 (53.3, 78.8)
Sp%, (95% CI)	67.7 (51.3, 84.2)	80.6 (66.7, 94.6)	80.6 (66.7, 94.6)
PPV%, (95% CI)	80.0 (68.9, 91.1)	86.4 (76.2, 96.5)	85.4 (74.5, 96.2)
NPV%, (95% CI)	61.8 (45.4, 78.1)	62.5 (47.5, 77.5)	58.1 (43.4, 72.9)
PLR (95% CI)	2.34 (1.37, 3.99)	3.7 (1.77, 7.75)	3.4 (1.62, 7.18)
NLR (95% CI)	0.36 (0.21, 0.61)	0.4 (0.22, 0.55)	0.4 (0.28, 0.64)

Note: ACC= accuracy; Se = sensitivity; Sp = specificity; PPV = positive predictive value; NPV = negative predictive value; PLR = positive likelihood ratio; NLR = negative likelihood ratio; lactate and D-dimer combination variable: y′= (−1.252) + 0.192 × lactate + 0.111 × D-dimer, probability=1/(1+e^−y′^)

**Table 4:** Hosmer-Lemeshow goodness-of-fit test for calibrating studied variables

	Lactate		D-dimer		Lactate and D-dimer	

	*χ^2^*(df)	*P*-value	*χ^2^*(df)	*P*-value	*χ*^2^(df)	*P*-value
Age group						
< 5 years old	4.976 (8)	0.760	6.928 (8)	0.545	8.150 (8)	0.419
≥ 5 years old	3.289 (8)	0.914	7.445 (8)	0.489	4.692 (8)	0.789
Gender						
Male	11.914 (8)	0.155	7.476 (8)	0.486	6.146 (8)	0.631
Female	8.609 (8)	0.376	3.638 (7)	0.820	0.971 (7)	0.995
Main diagnosis						
Sepsis	5.018 (8)	0.756	6.815 (8)	0.557	8.941 (8)	0.347
Non-sepsis	6.698 (8)	0.569	6.988 (8)	0.538	8.045 (8)	0.429
Overall	12.442 (8)	0.133	9.167 (8)	0.328	2.899 (8)	0.941
